# Tg737 signaling is required for hypoxia-enhanced invasion and migration of hepatoma cells

**DOI:** 10.1186/1756-9966-31-75

**Published:** 2012-09-13

**Authors:** Nan You, Weihui Liu, Lijun Tang, Xiao Zhong, Ru Ji, Ning Zhang, Desheng Wang, Yong He, Kefeng Dou, Kaishan Tao

**Affiliations:** 1 Department of Hepatobiliary Surgery, Xijing Hospital, Fourth Military Medical University, Xi’an 710032, China; 2 PLA Center of General Surgery; General Hospital of Chengdu Army Region, Chengdu, 610083, China; 3 Department of Urology, Xinqiao Hospital, Third Military Medical University, Chongqing, 400038, PR China

**Keywords:** Tg737, Hepatocellular carcinoma (HCC), Hypoxia, Migration, Invasion

## Abstract

**Background:**

Although hypoxia is known to promote hepatoma cell invasion and migration, little is known regarding the molecular mechanisms of this process. Our previous research showed that loss of Tg737 is associated with hepatoma cell invasion and migration; therefore, we hypothesized that the Tg737 signal might be required for hypoxia-enhanced invasion and migration.

**Methods:**

We established in vitro normoxic or hypoxic models to investigate the role of Tg737 in the hypoxia-enhanced invasion and migration of hepatoma cells. The hepatoma cell lines HepG2 and MHCC97-H were subjected to normoxic or hypoxic conditions, and the cell adhesion, invasion, and migration capabilities were tested. The expression of Tg737 under normoxia or hypoxia was detected using western blot assays; cell viability was determined using flow cytometry. Furthermore, we created HepG2 and MHCC97-H cells that over expressed Tg737 prior to incubation under hypoxia and investigated their metastatic characteristics. Finally, we analyzed the involvement of critical molecular events known to regulate invasion and migration.

**Results:**

In this study, Tg737 expression was significantly inhibited in HepG2 and MHCC97-H cells following exposure to hypoxia. The down regulation of Tg737 expression corresponded to significantly decreased adhesion and increased invasion and migration. Hypoxia also decreased the expression/secretion of polycystin-1, increased the secretion of interleukin-8 (IL-8), and increased the levels of active and total transforming growth factor β 1 (TGF-β1), critical regulators of cell invasion and migration. Moreover, the decrease in adhesiveness and the increase in the invasive and migratory capacities of hypoxia-treated hepatoma cells were attenuated by pcDNA3.1-Tg737 transfection prior to hypoxia. Finally, following the up regulation of Tg737, the expression/secretion of polycystin-1 increased, and the secretion of IL-8 and the levels of active and total TGF-β1 decreased correspondingly.

**Conclusions:**

These data provide evidence that Tg737 contributes to hypoxia-induced invasion and migration, partially through the polycystin-1, IL-8, and TGF-β1 pathway. Taken together, this work suggests that Tg737 is involved in the invasion and migration of hepatoma cells under hypoxia, with the involvement of the polycystin-1, IL-8, and TGF-β1 signaling pathway. Tg737 is a potential therapeutic target for inhibiting the high invasion and migration potential of hepatoma cells in hypoxic regions.

## Background

Hepatocellular carcinoma (HCC) is among the most common malignancies, with an increasing incidence in China [1]. Despite surgical and locoregional therapies, the prognosis remains poor because of local invasion and the high rate of intra-hepatic and distant metastases after resection or transplantation [2]. Invasion and metastasis have become major challenges that must be overcome for the effective treatment of HCC. Thus, advances in treatments for this disease are likely to develop from a better understanding of the mechanisms of invasion and metastasis.

In HCC, tissue oxygenation measurements have revealed large areas of hypoxic tissue, and the expression of hypoxic markers has been correlated with rapid invasion and metastasis, short overall survival, and short time to recurrence. It has been established that hypoxia is an important micro-environmental factor in prompting tumor invasion and metastasis [3]. Under hypoxic conditions, cells invasion and metastasis involve several sequential steps and a large number of altered molecules (such as cytokines, chemokines and their receptors, and growth factors) [4,5]. However, the precise and key molecular events that initiate this crucial turning point remain unknown, and this knowledge gap can lead to delays in diagnosis and poor treatment.

The Tg737 gene (GenBank: U203621) is an important tumor suppressor gene in HCC [6]. In a previous investigation, we showed that loss of heterozygosity (LOH) of the Tg737 gene at markers SHGC-57879 and G64212 closely correlates with tumor node metastasis (TNM) stage and with HCC metastasis, indicating that these two markers can be detected independently and used to predict tumor stage and metastasis in HCC patients [7]. We further found that reduced expression of Tg737 greatly promotes cell invasion and hepatocarcinogenesis of fetal liver stem/progenitor cells (FLSPCs) [8]. Based on the above findings, we hypothesized that Tg737 might play an important role in HCC invasion and metastasis. However, whether Tg737 plays a role in hypoxia-induced invasion and migration of HCC cells has not been reported. It is of paramount importance to gain this knowledge, not only to increase our understanding of tumor biology but also to permit the development of specific therapies that effectively target HCC.

The aim of this study was to investigate whether Tg737 correlates with hypoxia-induced HCC invasion and metastasis and to determine the underlying mechanisms of invasion and metastasis under hypoxic conditions. As we speculated, following exposure of HCC cells to hypoxia, downregulation of Tg737 is required for a significant increase in invasion and migration in vitro via the downregulation of polycystin-1 expression/secretion and the upregulation of interleukin-8 (IL-8) secretion and active and total transforming growth factor β 1 (TGF-β1) levels. To our knowledge, this is the first report demonstrating that Tg737 contributes to hypoxia-induced invasion and migration in HCC cells. The results of this research indicate that Tg737 may play a role in HCC gene therapy and should be investigated further.

## Materials and methods

### Cell line and culture condition

HepG2 and MHCC97-H cells (maintained in our laboratory, originally obtained from the Cell Bank of Type Culture Collection of the Chinese Academy of Sciences), were cultured in Dulbecco's Modified Eagle Medium (DMEM) supplemented with 10% fetal bovine serum (FBS; Invitrogen, Carlsbad, CA, USA), 100 IU/ml penicillin, 400 IU/L trypsin, and 100 μg/ml streptomycin and were plated in 75-cm^2^ flasks and cultured at 37°C with 5% CO_2_ and 95% humidified air. The medium was changed every 2 days. In all subsequent related experiments, the HepG2 and MHCC97-H cells were treated with medium supplemented with 1% FBS, unless otherwise noted. For the incubation of cells under hypoxic conditions, the cells were exposed to 1% O_2_ with 5% CO_2_ at 37°C for the indicated times.

### Annexin V/propidium iodide (PI) assay

To exclude the possibility of apoptosis-related effects in subsequent experiments, Annexin V/propidium iodide assays were performed. After 18 h of incubation with medium supplemented with 1% FBS under normoxic or hypoxic conditions at 37°C, the cells were harvested, washed in cold phosphate-buffered saline (PBS), incubated for 15 min with fluorescein-conjugated Annexin V and PI and analyzed using flow cytometry. The cells incubated with medium supplemented with 10% FBS under normoxic conditions were also analyzed.

### Adhesion assay

An adhesion assay was performed in 12-well plates as described elsewhere [9]. After 10 h of incubation with medium supplemented with 1% FBS at 37°C under normoxic or hypoxic conditions, the cells were harvested, resuspended (1 × 10^5^ in 1.5 ml of DMEM supplemented with 1% FBS), plated onto collagen surfaces, and allowed to adhere for 2 h, consistent with the previous conditions (normoxia or hypoxia). The unbound cells were removed by washing twice with PBS, and the adherent cells were counted under a microscope at 200× magnification from 10 random fields in each well. Each experiment was performed in triplicate.

### Cell invasion and migration assays

Cell migration was assayed using transwells with 8-μm pore filters (Costar, MA, USA). The lower chamber was filled with DMEM supplemented with 10% FBS and 5 μg/ml of fibronectin (Sigma, St. Louis, MO, USA), and 2 × 10^4^ cells in 0.5 ml of media supplemented with 1% FBS were loaded into the upper chamber. After 12 h of normoxic or hypoxic treatment, the cells that migrated to the bottom surface of the membrane were fixed with 4% formaldehyde and stained with 0.5% crystal violet dye. The cells on the top surface of the membrane were removed by wiping the surface with a cotton swab. The numbers of migrated cells were counted at 200× magnification from 10 different microscopic fields. For the Matrigel invasion assay, the procedures were the same as described above, except that the transwell membrane was coated with 500 ng/μl of Matrigel (BD, CA, USA).

### Protein extraction and western blot analyses

After being cultured in DMEM supplemented with 1% FBS under normoxic or hypoxic conditions for 12 h, the cells were processed for protein extraction, and western blot assays were performed according to the published method [10]. The primary antibodies were anti-glyceraldehyde-3-phosphate dehydrogenase (GAPDH) (diluted 1:400, Santa Cruz Biotechnologies, Santa Cruz, CA, USA) and anti-Tg737 (diluted 1:600, Abnova, Taipei, Taiwan). The grayscale values of each band on the blots were measured using BandScan 4.3. The cells incubated with medium supplemented with 10% FBS under normoxic conditions were also analyzed.

### Construction of the targeting vector

The pcDNA3.1-Tg737 plasmid was commercially constructed by the GeneChem Company (Shanghai, China) and was used for transient transfections. Briefly, the Tg737 coding sequence was amplified using the polymerase chain reaction (PCR) technique. Total RNA from normal human liver tissue was isolated with Trizol (Invitrogen). Normal human liver tissue was obtained from patients who consented to the procedure during a laparotomy and hepatic resection. The tissues were acquired following approval by the local medical research ethics committee at Xijing Hospital, the Fourth Military Medical University, Xi’an, China. A High Fidelity PrimeScript reverse transcription PCR kit (TaKaRa, Dalian, China) was used to synthesize cDNA according to the manufacturer’s protocol. The PCR was performed with the primer set P1, 5’-TCCGCTCGAGATGAAATTCACAAACACTAAGGTAC-3’ (forward) and P2, 5’-ATGGGGTACCTTATTCTGGAAGCAAATCATCTCCT-3’ (reverse), containing XhoI and KpnI sites, respectively, using the obtained cDNA as a template. The following cycling conditions were used: initial denaturation at 94°C for 5 min; 30 cycles of denaturation at 94°C for 10 s, annealing at 55°C for 30 s, and extension at 72°C for 2 min; and a final extension at 72°C for 10 min. After digestion using XhoI and KpnI enzymes, the PCR product was cloned into the pcDNA3.1 (−) vector (GnenChem, Shanghai, China) digested using the same enzymes; the resultant recombinant plasmid was designated pcDNA3.1-Tg737.

### Transient transfection and cell adhesion, invasion and migration assays

The pcDNA3.1-Tg737 plasmid was transiently transfected into HepG2 and MHCC97-H cells using LipofectamineTM 2000 (Invitrogen). All of the procedures were performed according to the manufacturer’s instructions. The cells transfected with pcDNA3.1 (−), those incubated with LipofectamineTM 2000 alone and those without plasmid transfection were established as controls. Six hours after transfection, transiently pcDNA3.1-Tg737-transfection cells and controls were subjected to the analyses described above. In brief, the cells were incubated with fresh DMEM (1% FBS) for 12 h under hypoxia and were then subjected to western blot analysis for Tg737 expression. After 10 h of incubation under hypoxia, the cells underwent an adhesion assay. Furthermore, the cells (approximately 2 × 10^4^ cells) in 0.5 ml of media supplemented with 1% FBS were plated into the top chamber of a transwell and were incubated for 12 h under hypoxic conditions for the migration and invasion assays. After 12 h of incubation under hypoxia, Annexin V/propidium iodide assays were also performed to exclude apoptosis-related effects.

### Western blot assay for polycystin-1

To measure the polycystin-1 expression levels of the different cells (indicated in the Results and Figure Legends sections), western blot assays were performed using the techniques described above. The primary antibodies used were anti-polycystin-1 (diluted 1:600, Santa Cruz) and anti-GAPDH (diluted 1:400, Santa Cruz).

### Enzyme-linked immunosorbent assay (ELISA)

For quantification of polycystin-1, IL-8 and TGF-β1 protein secretion by different cells, culture medium was collected and centrifuged at 6000 r/min for 10 min. The supernatant was used for determination of protein secretion with ELISA kits (Cusabio, Wuhan, China) according to the manufacturer’s protocol. The antibodies used in the TGF-β1 ELISA kit are only able to detect TGF-β1 in its active form; thus, the samples were activated by acidification before ELISA to determine the amount of total TGF-β1.

### Statistical analysis

SPSS software, version 14.0, was used for all statistical evaluations. The data are presented as the means ± standard errors of the mean for separate experiments (n ≥ 3, where n represents the number of independent experiments). The data were analyzed for significance using a one-way ANOVA; *P* < 0.05 was considered significant.

## Results

### Hypoxia reduced HCC cell adhesion and facilitated invasion and migration

**Figure 1:**
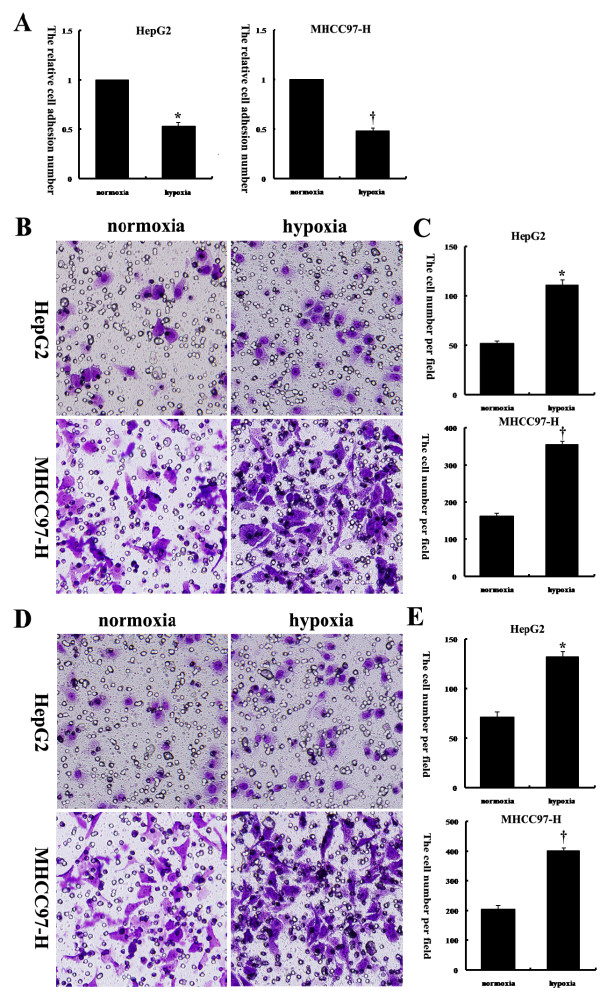
**Hypoxia reduced HepG2 and MHCC97-H cell adhesion and facilitated invasion and migration.** (**A**) An adhesion assay was performed with HCC cells on collagen I-coated plates. The relative cell adhesion number in each group is reflected in the column chart. The values of the normoxia-treated cells were set at 1. (**B, C**) Matrigel invasion assays of HepG2 and MHCC97-H cells were performed under normoxic and hypoxic conditions; the quantified data are shown in the diagram. (**D, E**) Transwell migration assays of HepG2 and MHCC97-H cells were performed under normoxic and hypoxic conditions; the numbers of cells are shown in the diagram. ^*^, *P* < 0.05 compared to normoxia-treated HepG2 cells; ^†^, *P* < 0.05 compared to normoxia-treated MHCC97-H cells. Original magnification: 200× (B, D).

**Figure 2:**
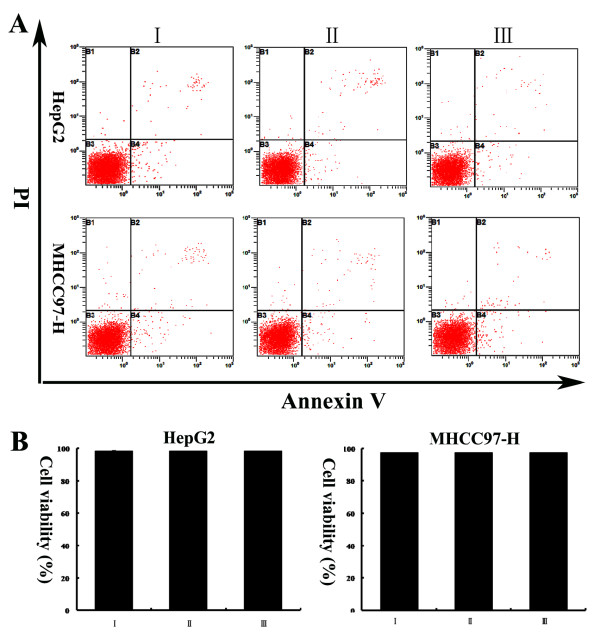
(**A) Representative dot plots showing the effects of low-serum medium under normoxic or hypoxic conditions on HepG2 and MHCC97-H cell apoptosis.** The cultured cells were treated for the indicated time periods and then stained with FITC-conjugated Annexin V and PI. (**B**) The percentage of viable cells in each group is reflected in the column chart. I: cells incubated with medium supplemented with 10% FBS under normoxia; II: cells incubated with medium supplemented with 1% FBS under normoxia; III: cells incubated with medium supplemented with 1% FBS under hypoxia.

To examine the effects of hypoxia on HCC cell adhesion, migration, and invasion, two human HCC cell lines, HepG2 and MHCC97-H, were exposed to either normoxia or hypoxia under the same media conditions. An adhesion assay revealed that exposure of these two HCC cell lines to hypoxic conditions decreased their capacity to adhere to collagen (Figure 1A). Next, HCC cell migration through a microporous membrane and invasion through an extracellular matrix were assessed under normoxic and hypoxic conditions. It was observed that exposure of these two HCC cell lines to hypoxic conditions resulted in significant increases in invasion (Figure 1B and C) and migration (Figure 1D and E) in vitro. To exclude the effects on cell viability after treatment with low-serum medium under normoxic or hypoxic conditions, we performed Annexin V assays. We found that over the course of the experiment, the treatment of HepG2 and MHCC97-H cells with low-serum medium under normoxic or hypoxic conditions did not significantly affect cell viability in vitro (Figure 2A and B).

### Hypoxia induced the downregulation of Tg737 expression in HCC cells

**Figure 3:**
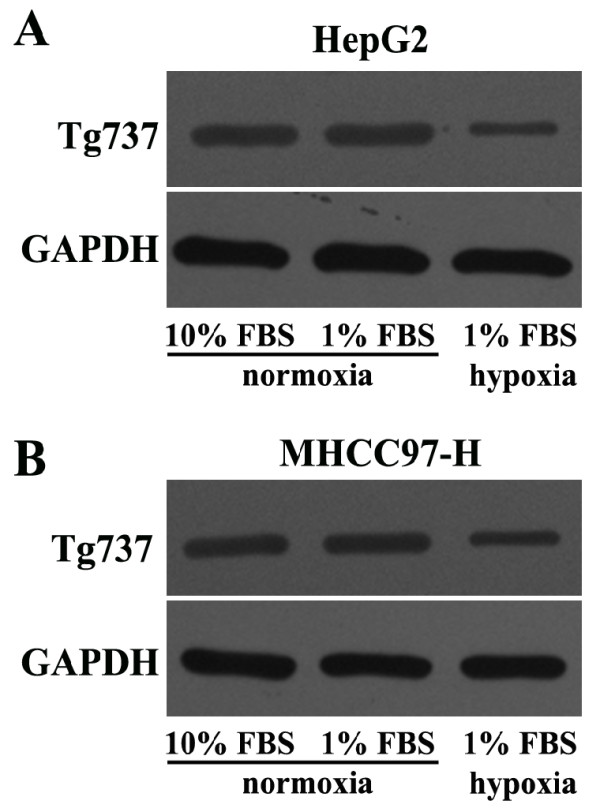
**Hypoxia inhibited Tg737 expression in HepG2 and MHCC97-H cells. **Western blot assay for Tg737 was performed; GAPDH was used as a control.

To determine whether Tg737 played a role in the decreased adhesion and increased invasion and migration capacity of hypoxia-treated HCC cells, western blot assays were used to detect Tg737 expression. Under the same media conditions, the exposure of HepG2 and MHCC97-H to hypoxia led to a significant decrease in Tg737 expression levels compared to cells exposed to normoxia (Figure 3A and B). However, the treatment of HepG2 and MHCC97-H cells with low-serum medium under normoxia did not significantly affect Tg737 expression.

### pcDNA3.1-Tg737 transfection prior to incubation in hypoxia facilitated HCC cell adhesion and attenuated cell migration and invasion

Following confirmation of the relationships among changes in adhesion, invasion and migration capacity and the downregulation of Tg737 expression in hypoxia-treated HCC cells, we wished to further clarify whether Tg737 played a role in this process. The Tg737 DNA fragment was inserted into the pcDNA3.1 (−) vector. The data in Additional file 1 and Additional file 2 in the Supplemental Data section confirmed that the recombinant plasmid contained the correct, full-nucleotide sequence of the Tg737 gene.

**Figure 4:**
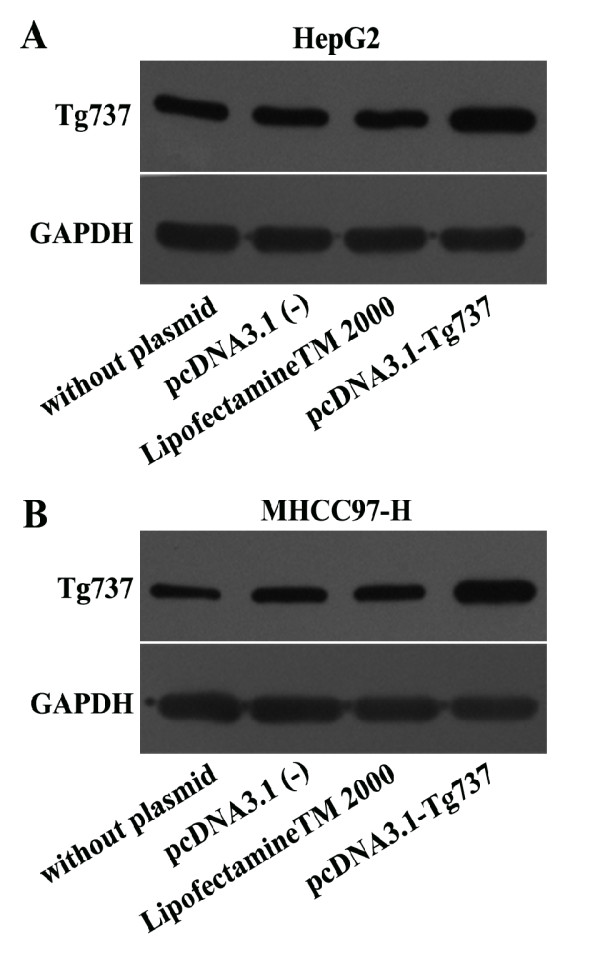
**Western blot assay was performed to determine the expression levels of Tg737 in the different cells.** The HepG2 and MHCC97-H cells were transiently transfected with the pcDNA3.1-Tg737 plasmid. To exclude liposome/vector-related effects, HepG2 and MHCC97-H cells transfected with pcDNA3.1 (−) or incubated with LipofectamineTM 2000 alone were used as controls. HepG2 and MHCC97-H cells without plasmid transfection also served as blank controls. The cells were incubated with fresh DMEM (1% FBS) for 12 h under hypoxia, then lysed and subjected to immunoblot analysis.

**Figure 5:**
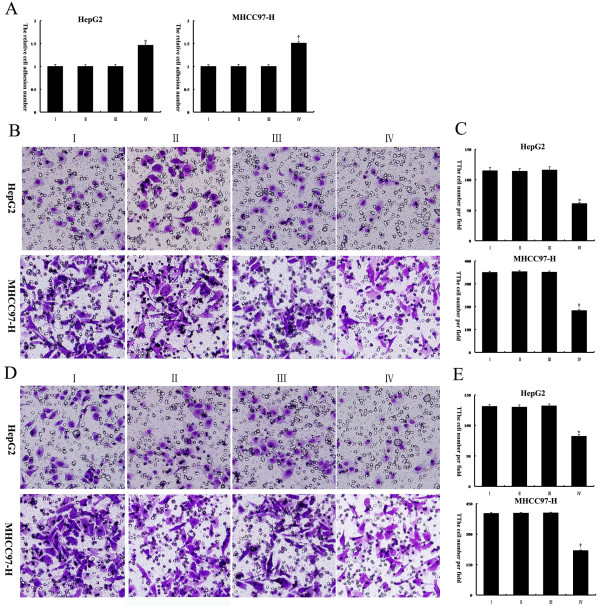
**The effects of Tg737 over expression on cell adhesion, invasion, and migration in hypoxia-treated HCC cells.** HepG2 and MHCC97-H cells were treated as detailed in the legend to Figure 4. (**A**) An adhesion assay was used to evaluate the effects of Tg737 on adhesion. The values of the cells incubated with medium supplemented with 10% FBS under normoxia were set at 1. (**B, C**) The stained membrane after cell invasion demonstrated that Tg737 over expression in HepG2 and MHCC97-H cells led to significantly attenuated cell invasion under hypoxic conditions compared to cells without plasmid transfection under hypoxic conditions. The data are presented as the number of invading cells for each group. (**D, E**) The effects of Tg737 over expression on the migration capacity of hypoxia-treated HCC cells were investigated using a transwell migration assay. The data are presented as the number of migrated cells for each group. I: cells without plasmid transfection; II: cells transfected with pcDNA3.1 (−); III: cells incubated with LipofectamineTM 2000; IV: cells transfected with pcDNA3.1-Tg737. ^*^, *P* < 0.05 compared to the HepG2 controls; ^†^, *P* < 0.05 compared to the MHCC97 controls. Original magnification: 200× (**B, D**).

**Figure 6:**
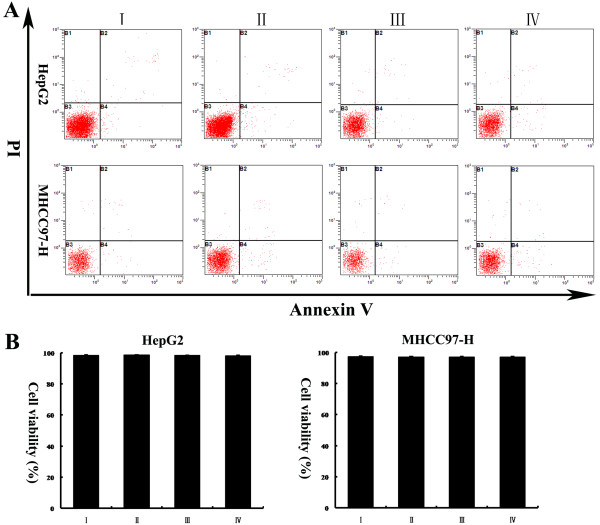
**(A, B) HepG2 and MHCC97-H cells were treated as detailed in the legend to Figure**4**.** Annexin V assays revealed that the cell viability of HepG2 and MHCC97-H cells transfected with pcDNA3.1-Tg737 and further incubated with fresh DMEM (1% FBS) for 12 h under hypoxia were not significantly different from cells without plasmid transfection. The data from HepG2 and MHCC97-H cells transfected with pcDNA3.1 (−) or incubated with LipofectamineTM 2000 excluded any liposome/pEGFP-C1-related effects on cell viability.I: cells without plasmid transfection; II: cells transfected with pcDNA3.1 (−); III: cells incubated with LipofectamineTM 2000; IV: cells transfected with pcDNA3.1-Tg737.

The pcDNA3.1-Tg737 plasmids were transiently transfected into HepG2 and MHCC97-H cells; the Tg737 protein levels were then determined, and the adhesion, invasion and migration experiments were repeated under hypoxia. As shown in Figure 4A and B, when pcDNA3.1-Tg737-transfection cells and cells without plasmid transfection were incubated with DMEM supplemented with 1% FBS for 12 h under hypoxia, western blot analysis showed an increase in the Tg737 protein in pcDNA3.1-Tg737-transfection cells, compared to cells without plasmid transfection (n = 3, *p* < 0.05). These data indicated that although the cells were transfected with pcDNA3.1-Tg737 prior to incubation under hypoxia, the pcDNA3.1-Tg737 used in this study was effective in promoting the overexpression of the Tg737 gene in HepG2 and MHCC97-H cells. Furthermore, it was observed that under the same media conditions, the overexpression of Tg737 in HepG2 and MHCC97-H cells significantly facilitated cell adhesion and attenuated cell invasion and migration under hypoxic conditions compared to cells without plasmid transfection under hypoxic conditions (Figure 5A-E). To confirm that the effects of Tg737 overexpression on the facilitation of HCC cell adhesion and on the attenuation of invasion and migration under hypoxic conditions were not due to decreased cell viability resulting from transfection with pcDNA3.1-Tg737, we assessed the effect of pcDNA3.1-Tg737 transfection on cell viability using Annexin V assays. As shown in Figure 6A and B, the transfection of pcDNA3.1-Tg737 and subsequent hypoxia treatment did not affect cell viability compared to cells without plasmid transfection under hypoxic conditions. To exclude liposome/pcDNA3.1 (−)-related effects on our study, we also analyzed cell viability and Tg737 expression, adhesion, invasion and migration in HepG2 and MHCC97 cells transfected with pcDNA3.1 (−) or incubated with LipofectamineTM 2000 prior to incubation in hypoxia. Cell viability, Tg737 protein levels, and the adhesion, migration and invasion of these cells exhibited no significant differences compared to cells without plasmid transfection (n = 3, *P* > 0.05). The results suggest that liposome/pcDNA3.1 (−) had no effects in our study.

### Polycystin-1, IL-8, and TGF-β1 were associated with the contribution of Tg737 to hypoxia-induced adhesion, migration, and invasion

**Figure 7:**
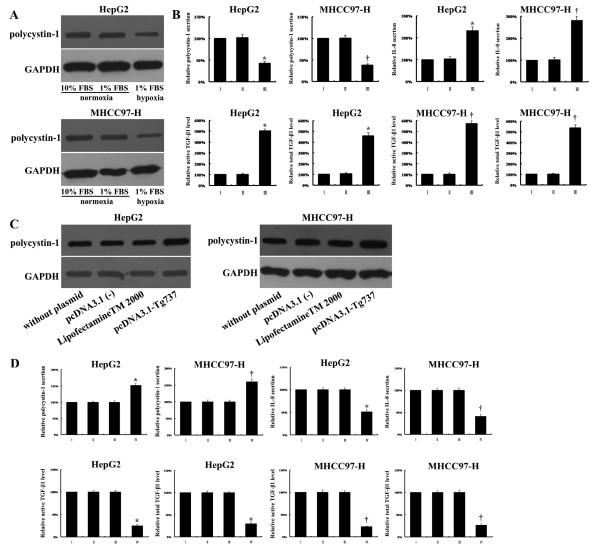
(**A) The cells were harvested with ice-cold PBS and lysed, and polycystin-1 levels were determined using western blot analysis.** The expression levels of polycystin-1 in HepG2 and MHCC97-H cells were decreased in response to hypoxia. (**B**) The cells were subjected to ELISA for analysis of the secretion of polycystin-1, IL-8 and TGF-β1. I: cells incubated with medium supplemented with 10% FBS under normoxia; II: cells incubated with medium supplemented with 1% FBS under normoxia; III: cells incubated with medium supplemented with 1% FBS under hypoxia. The values of the cells incubated with medium supplemented with 10% FBS under normoxia were set at 100%. (**C**) Western blot assays showed increased polycystin-1 protein expression levels in hypoxia-cultured HepG2 and MHCC97-H cells transfected with pcDNA3.1-Tg737. (**D**) ELISA revealed increased polycystin-1 secretion and decreased IL-8 secretion and decreased active and total TGF-β1 levels in hypoxia-cultured HepG2 and MHCC97-H cells transfected with pcDNA3.1-Tg737. The values of cells without plasmid transfection were set at 100%. I: cells without plasmid transfection; II: cells transfected with pcDNA3.1 (−); III: cells incubated with LipofectamineTM 2000; IV: cells transfected with pcDNA3.1-Tg737. ^*^, *P* < 0.05 compared to the HepG2 controls; ^†^, *P* < 0.05 compared to the MHCC97 controls.

To further explore the mechanism of action of Tg737 in hypoxia-induced adhesion, migration, and invasion in HCC cells, we examined the effects of Tg737 on the expression/secretion of polycystin-1 and the secretion of IL-8 and TGF-β1, critical regulators of cell invasion and migration. Our data indicated that polycystin-1 protein expression/secretion was downregulated, whereas IL-8 secretion and the active and total TGF-β1 levels were increased by hypoxia treatment. These expression patterns were consistent with Tg737 downregulation compared to normoxia-treated cells. Furthermore, the levels of polycystin-1, IL-8, and TGF-β1 (active and total) in hypoxia-treated HepG2 and MHCC97-H cells could be recovered in both lines by transfection with pcDNA3.1-Tg737. The levels of polycystin-1, IL-8, and TGF-β1 (active and total) were altered with the restored expression of Tg737 (Figure 7A-D). Taken together, these results demonstrated that Tg737 regulated hypoxia-induced adhesion and that migration and invasion capabilities were partially mediated by polycystin-1, IL-8 and, TGF-β1 protein levels, possibly leading to subsequent degradation of the extracellular matrix.

## Discussion

The outcomes for patients with HCC remain dismal, although a great deal has been learned regarding the disease over the past few decades. The capacity of cancer cells to invade and metastasize to other locations in the body remains a major obstacle for improving the survival and prognosis of HCC patients. Despite extensive studies, a clear understanding of the mechanisms of the invasion and metastasis processes and of how tumor cells acquire these characteristic capabilities remains elusive [11,12].

One factor that may play an important role in invasion and metastasis is hypoxia, which commonly refers to a condition in tissues in which the oxygen pressure is less than 5–10 mmHg [13-15]. Hypoxia is a condition commonly found in a wide range of solid tumors including HCC, and it is often associated with a poor prognosis [16]. Recent studies have shown that HCC develops through cirrhosis induced by chronic liver injury. This chronic injury causes fibrogenesis, which demolishes the normal liver blood system. Damage to the liver blood system leads to a shortage of blood circulation in the liver and consequently leads to hypoxia. Moreover, the high proliferation of tumor cells also contributes to local hypoxia in HCC [17]. Oxygen starvation causes the cells to invade and migrate to distant sites and to colonize organs in which nutrients and space are less limited. Hypoxia potentially regulates each step of the invasion and metastasis process, from the initial epithelial-mesenchymal transition to organotropic colonization, suggesting a master regulator role for hypoxia in invasion and metastasis [18]. However, the molecular basis of this process is not well understood.

Hypoxia-induced increases in invasion and metastastic potential, and the dissemination of tumor cells from a primary tumor site to a secondary, distant site through the bloodstream or the lymphatic system are complex, multistep processes involving detachment from the matrix and neighboring cells, migration through the surrounding stroma, entry into the circulatory system, and finally arrest, extravasation, and growth at a secondary site. This multistep process is mediated by several mechanisms, including changes in gene expression, inactivation and/or the activation of genes, and enhanced genomic instability [19,20]. Several hypoxia-regulated genes have been identified thus far, including lysyl oxidase (LOX) [21], connective tissue growth factor (CTGF) [22], E-cadherin [23], CXCR4/SDF-1 [24], and migration inhibitory factor (MIF) [25]. However, although a general hypoxic gene signature that correlates with poor treatment outcomes has been defined, many invasion- and metastasis-related changes are tissue- and cell type-specific; therefore, relevant signatures can vary from one cell type to another [26]. Thus, further investigation is necessary for the identification of new, HCC-specific, hypoxia-regulated genes and for the determination of the corresponding signaling pathways. Interference with these specific genes to reduce hypoxia-induced invasion and metastasis could contribute to the development of anti-HCC therapies.

The Tg737 gene, a liver tumor suppressor gene of the tetratricopeptide repeat (TPR) family, plays an important role in liver carcinogenesis [6]. Significant down-regulation of the Tg737 gene has been observed in 59% of HCC tissues [27]. Furthermore, our preliminary studies have suggested that Tg737 is involved in HCC invasion and metastasis [7,8]. In this study, we presented the first evidence that the Tg737 gene has an important function in hypoxia-induced invasion and migration of HCC cells.

It has been established that cell-cell adhesion determines the polarity of cells, participates in the maintenance of the cell societies called tissues and is critical for carcinogenesis and cancer metastasis. Cell-cell adhesiveness is generally reduced in human cancers. Reduced cell-cell adhesiveness allows cancer cells to violate the local order, resulting in destruction of histological structure, which is the morphological hallmark of malignant tumors. Reduced intercellular adhesiveness is also essential for cancer invasion and metastasis [28]. Hypoxia could facilitate tumor cell detachment by reducing the expression of surface adhesion molecules and adhesion to the extracellular matrix [29]. As shown in our study, hypoxia-treated HepG2 and MHCC97-H cells exhibited reduced adhesion and increased invasion and migration compared to cells under normoxic conditions. We believe that the decreased adhesion of hypoxia-treated cells in our study may be a reduced intercellular adhesiveness, which may be a factor enabling malignant cells to escape hypoxia with the potential to form new foci of tumor growth. Our cell aggregation assay also showed that hypoxia inhibited hepatoma cell aggregation in our study (data not shown). To explore whether Tg737 is involved in invasion and migration induced by hypoxia, we examined the different expression levels of Tg737 under normoxic and hypoxic conditions. The data confirmed that hypoxia induced the downregulation of Tg737 expression in HCC cell lines. In addition, hypoxia induced changes in adhesion, and the migration and invasion capacities of HCC cells were abrogated by restoring Tg737 expression levels. Taken together, these results suggest that hypoxia may increase the invasion and migration of HCC cells in a Tg737-dependent manner.

The hypoxia-induced invasion and migration mediated by Tg737 is poorly understood. A hallmark of the invasion and migration of solid tumors is that this process requires cell-cell/matrix molecules that influence the adhesion, migration, and invasion of cancer cells [30]. Polycystin-1 is a large, plasma membrane receptor encoded by the PKD1 gene, which is mutated in autosomal-dominant polycystic kidney disease (ADPKD). Polycystin-1 is involved in several biological functions including proliferation, morphogenesis, and anti-apoptotic processes [31,32]. Moreover, polycystin-1 appears to be associated with the focal adhesion proteins talin, vinculin, FAK and paxillin [33]. Zhang et al. [9] also found that polycystin-1 influences the adhesion, migration, and invasion of cancer cells. As stated above, polycystin-1 is thought to be a cell adhesion molecule, possibly a member of the immunoglobulin superfamily of cell adhesion molecules. Furthermore, preliminary yeast 2-hybrid screens with Tg737 have identified several potential protein partners, including polycystin 1, catenin, P120 catenin, Snx1, and HNF4α [34]. Due to the importance of polycystin 1 in the adhesion, invasion and migration of cancer cells and as a potential protein partner of Tg737, we hypothesized that Tg737-mediated hypoxia-induced increases in invasion and migration require polycystin 1. As shown in our results, the expression of both Tg737 and polycystin 1 decreased after exposure of HCC cells to hypoxia. Moreover, the expression of polycystin 1 was restored under hypoxia by transfection of pcDNA3.1-Tg737. These data suggest that the effects of Tg737 on HCC cell migration and invasion under hypoxia may be at least partially mediated by the polycystin 1 pathway.

A large amount of evidence suggests that some cytokines and chemokines secreted by cancer cells are important modulators of migration and invasion. Among these, IL-8 and TGF-β1 have important roles in the invasion and metastasis of many types of tumors [35,36]. Furthermore, IL-8 and TGF-β1 signaling were recently investigated during the progression of ADPKD in PKD1 mutant models [37,38]. Therefore, we hypothesized that IL-8 and TGF-β1 may be closely associated with polycystin 1 and may be required for the contribution of Tg737 to hypoxia-induced invasion and migration in HCC cells. To clarify this hypothesis, we analyzed the secretion of IL-8 and TGF-β1 using ELISA and found that IL-8 secretion and the active and total TGF-β1 levels were increased in hypoxia-treated HepG2 and MHCC97-H cells. Furthermore, the secretion of IL-8 and both active and total TGF-β1 levels were restored by transfection of pcDNA3.1-Tg737 under hypoxia. These findings suggest that the Tg737-mediated hypoxia-induced increases in invasion and migration are associated with alterations in the secretion of IL-8 and TGF-β1. IL-8 and TGF-β1 may also be important intermediaries in the actions of Tg737 in HCC. However, the precise interactions between polycystin 1, IL-8, and TGF-β1 remain largely unexplored. Further identification of the exact interactions may provide more details regarding the mechanism of the effect of Tg737 on hypoxia-induced invasion and migration. In addition, using ELISA, we found that hypoxia decreased the secretion of polycystin-1, and pcDNA3.1-Tg737 restored polycystin 1 secretion under hypoxia. Future studies need to focus on the exact mechanism of polycystin 1, IL-8, and TGF-β1 actions in Tg737-mediated hypoxia-induced increases in invasion and migration.

Taken together, our observations suggest that Tg737 is involved in hypoxia-induced invasion and migration in HCC by regulating polycystin 1, IL-8, and TGF-β1. As is known, the best-characterized hypoxia response pathway is mediated by hypoxia-inducible factor (HIF). Hypoxia increases tumor glycolysis, angiogenesis and other survival responses, along with invasion and migration, by activating relevant genes through HIF [39]. It has been shown that the activation of HIF is not only induced by hypoxic conditions. Semenza [40] reviewed the mechanisms by which HIF-1 levels can be increased by dysfunctional tumor suppressor genes. However, the interaction between HIF and the Tg737 axis remains largely unexplored. Elucidating these details might provide more information regarding the mechanism of Tg737 effects on hypoxia-regulated invasion and migration.

## Conclusions

In this study, for the first time, we demonstrated that Tg737 plays a key role in hypoxia-mediated invasion and migration. The results of this study may be useful in designing novel therapeutic interventions that block hypoxia-dependent Tg737 expression and consequently block HCC invasion and metastasis.

## Abbreviations

ADPKD: Autosomal dominant polycystic kidney disease; CTGF: Connective tissue growth factor; DMEM: Dulbecco's Modified Eagle Medium; FBS: Fetal bovine serum; FLSPCs: Fetal liver stem/progenitor cells; GAPDH: Glyceraldehyde-3-phosphate dehydrogenase; HCC: Hepatocellular carcinoma; HIF: Hypoxia-inducible factor; IL-8: Interleukin-8; LOH: Loss of heterozygosity; MIF: Migration inhibitory factor; PBS: Phosphate-buffered saline; PCR: Polymerase chain reaction; PI: Propidium iodide; TGF-β1: Transforming growth factor β 1; TNM: Tumor node metastasis; TPR: Tetratricopeptide repeat.

## Misc

Nan You, Lijun Tang, Weihui Liu and Xiao Zhong contributed equally to this study.

## Competing interests

The authors declare that they have no competing interests.

## Authors’ contributions

All authors participated in the design, interpretation of the data and review of the manuscript. NY and WL performed the experiments and NY, WL and KT wrote the manuscript. All authors read and approved the final manuscript.

## Supplementary Material

Additional file 1 **The construction of the pcDNA3.1-Tg737 recombinant plasmid.** (**A**) The PCR results from the Tg737 gene are shown. Lane 1: marker; lane 2: Tg737 PCR products. (**B**) The identification of recombinant clones by PCR. Lane 1: negative control (ddH_2_O); lane 2: negative control (empty, self-ligated vector); lane 3: positive control (GAPDH); lane 4: marker; lanes 5–12: 1-8# transformation. Click here for file

Additional file 2 Sequence analysis. Click here for file
