# Supplementary figures and images for: Tg737 signaling is required for hypoxia-enhanced invasion and migration of hepatoma cells
Source: J Exp Clin Cancer Res. 2012 Sep 13;31(1):75. doi: 10.1186/1756-9966-31-75 (PMC3523075; doi:10.1186/1756-9966-31-75)

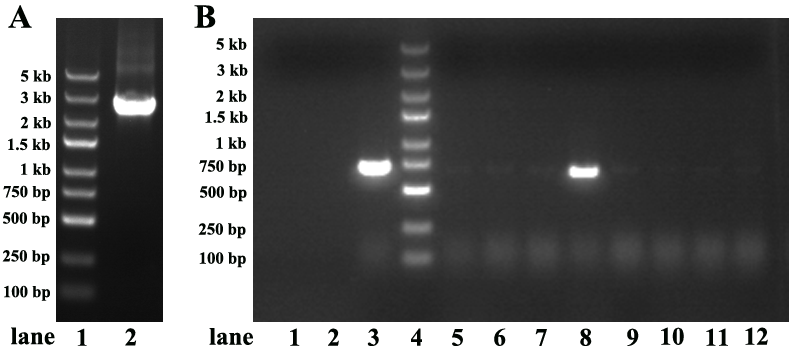

Supplement: Additional file 1 — The construction of the pcDNA3.1-Tg737 recombinant plasmid. (A) The PCR results from the Tg737 gene are shown. Lane 1: marker; lane 2: Tg737 PCR products. (B) The identification of recombinant clones by PCR. Lane 1: negative control (ddH2O); lane 2: negative control (empty, self-ligated vector); lane 3: positive control (GAPDH); lane 4: marker; lanes 5–12: 1-8# transformation. [file 1756-9966-31-75-S1.tiff]

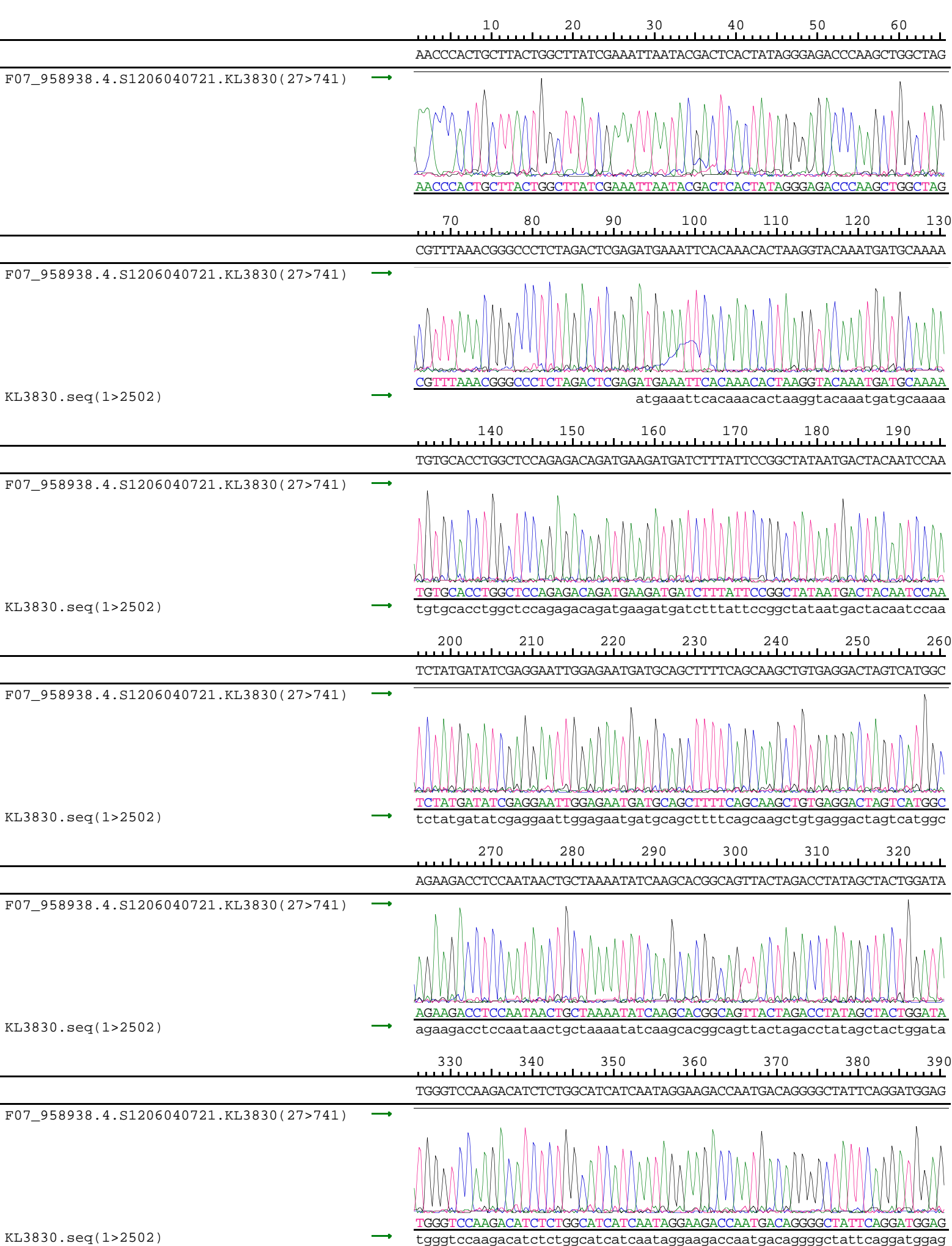

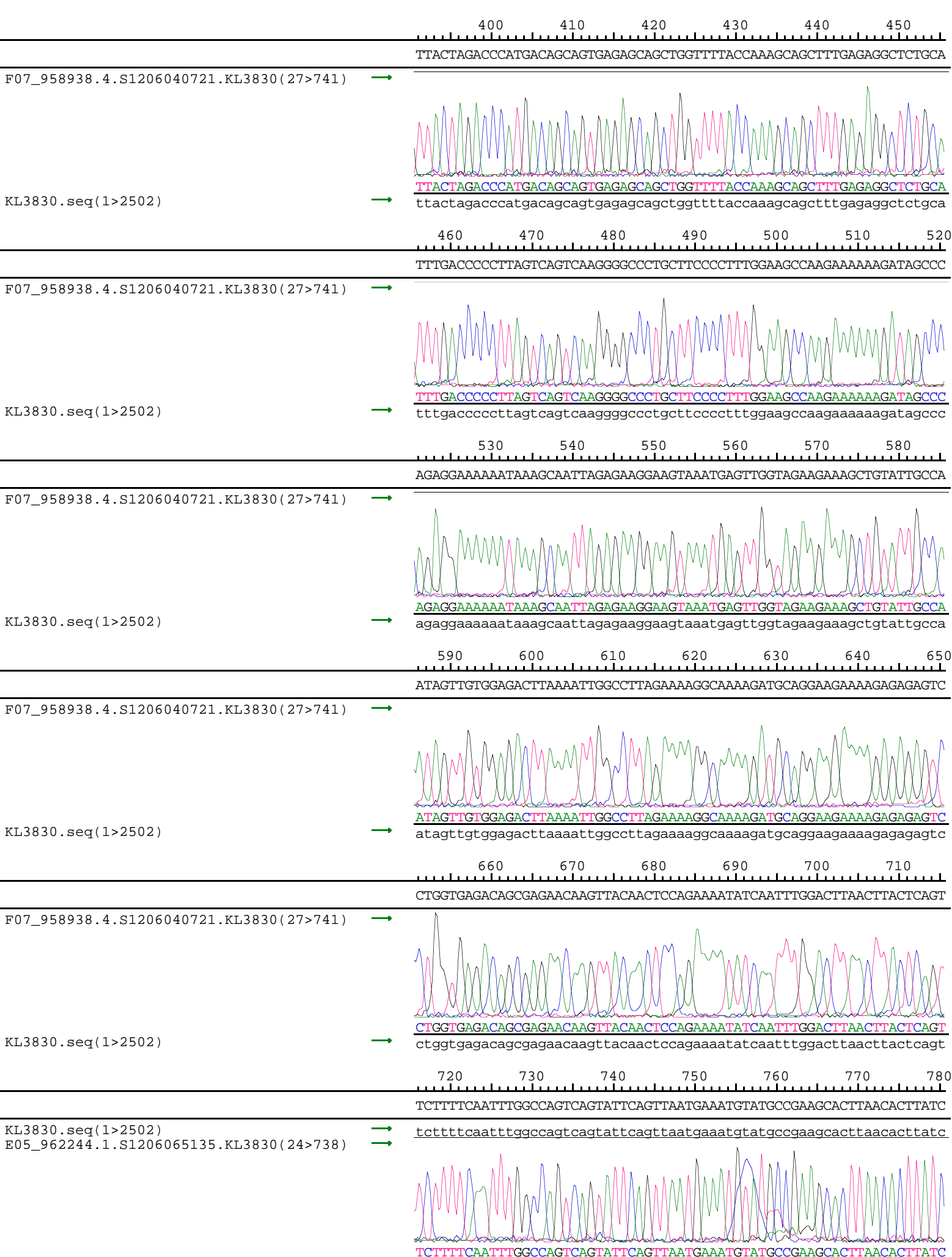

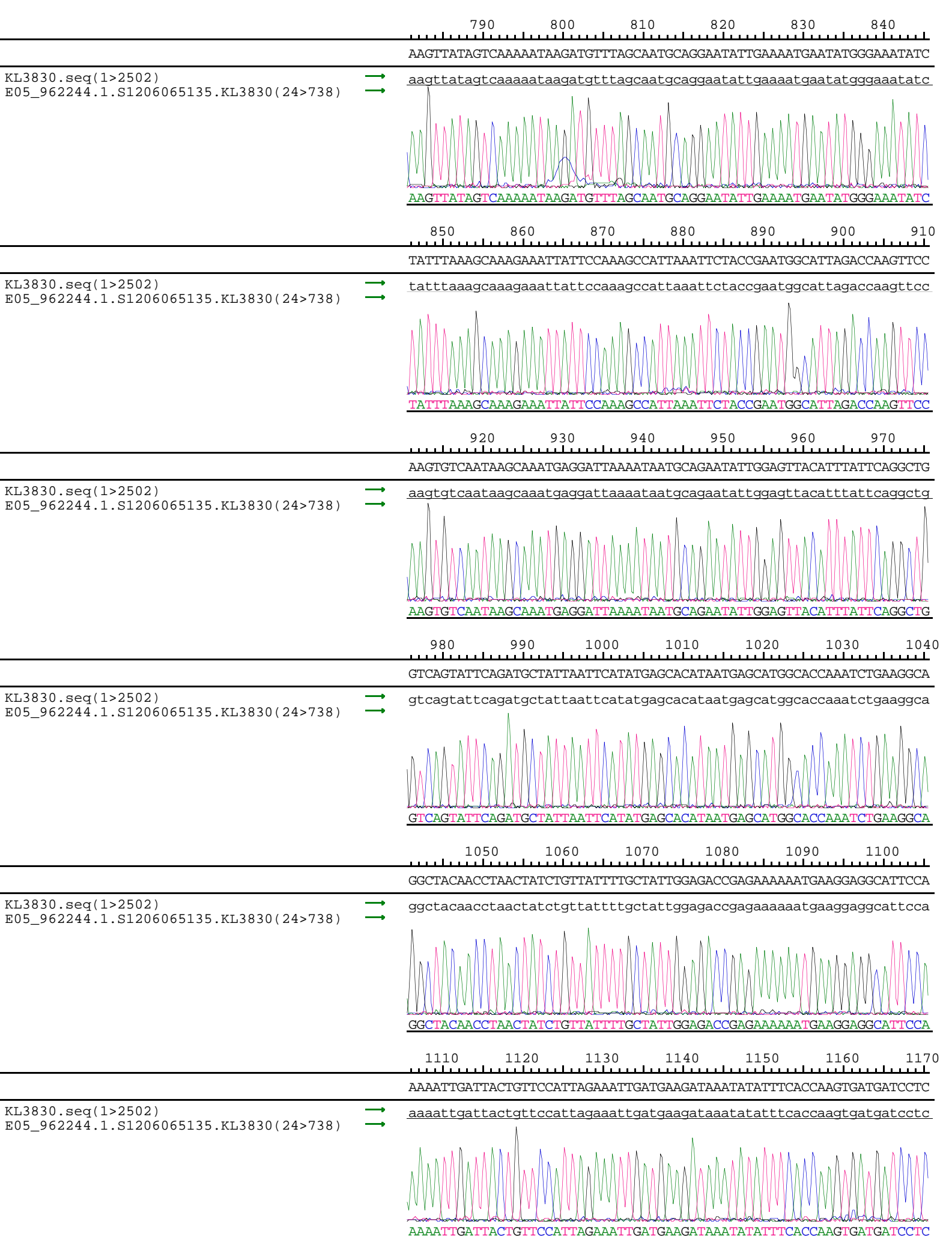

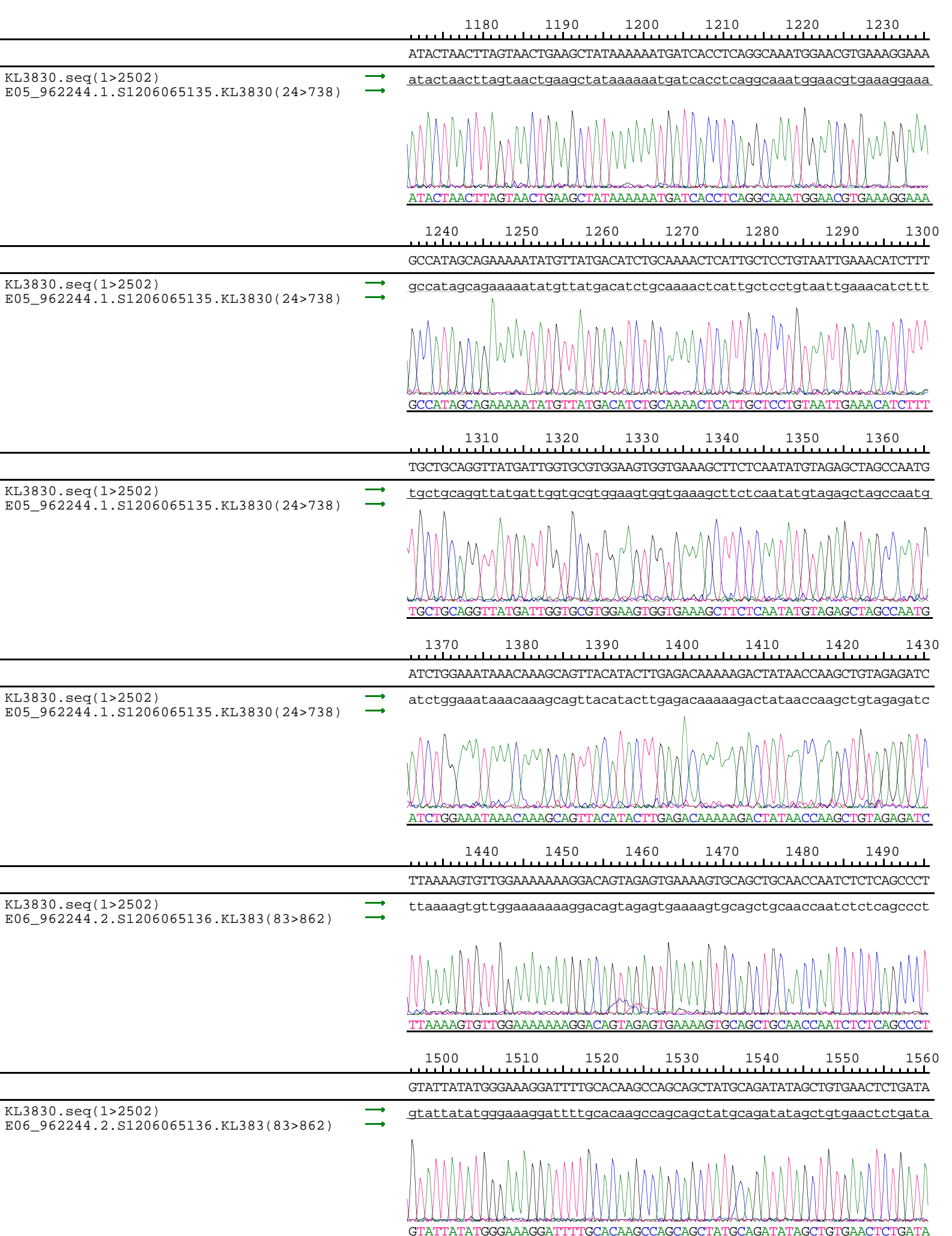

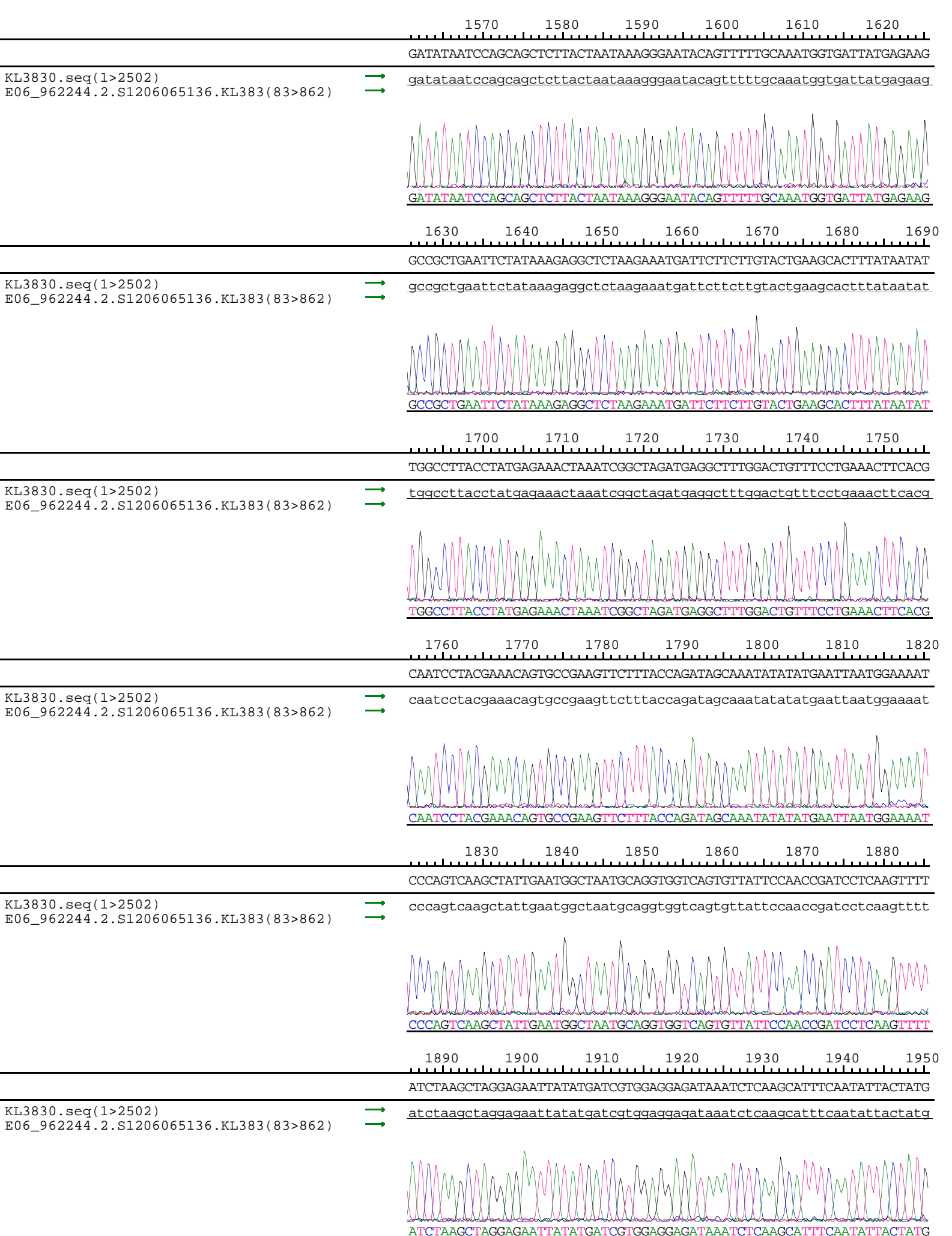

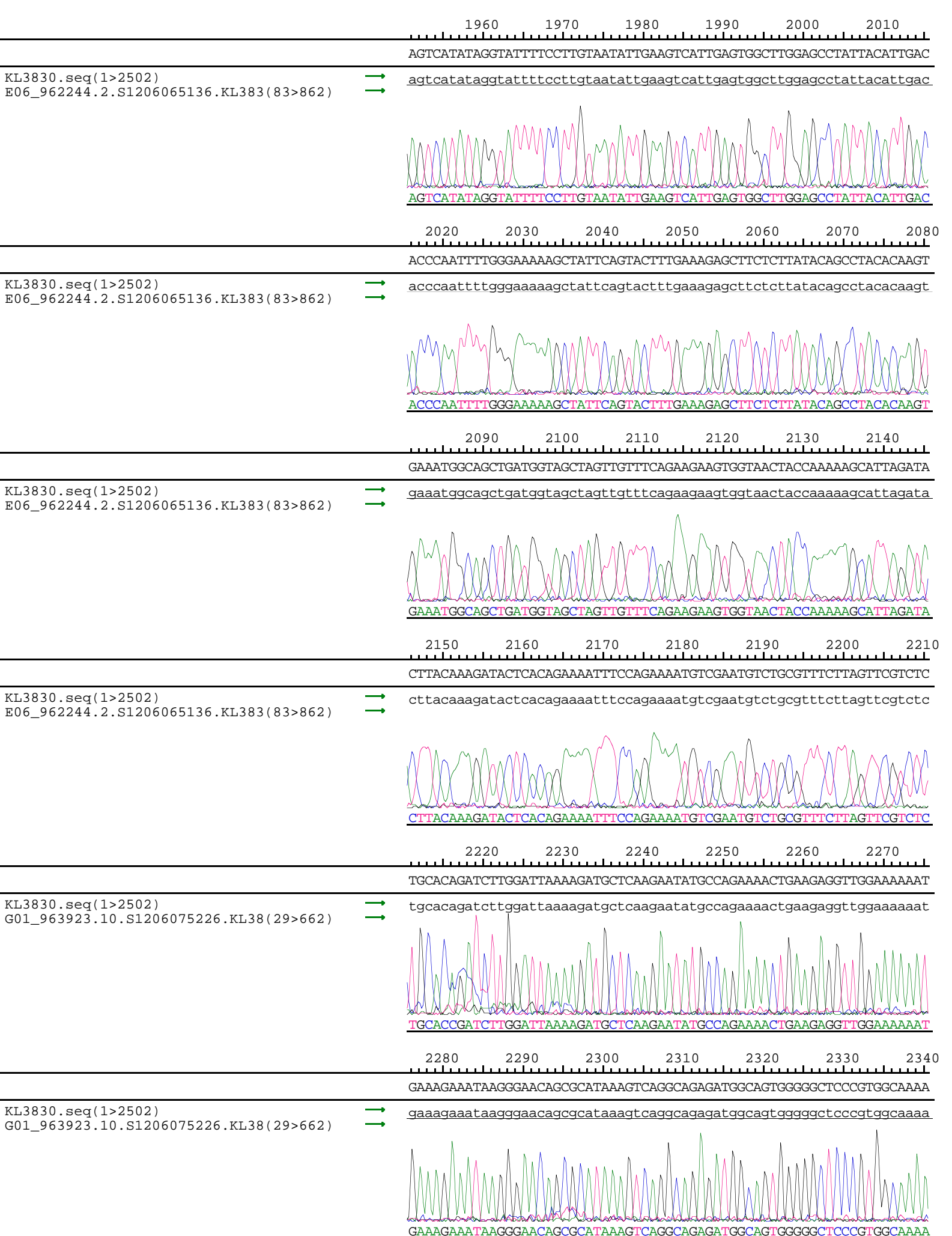

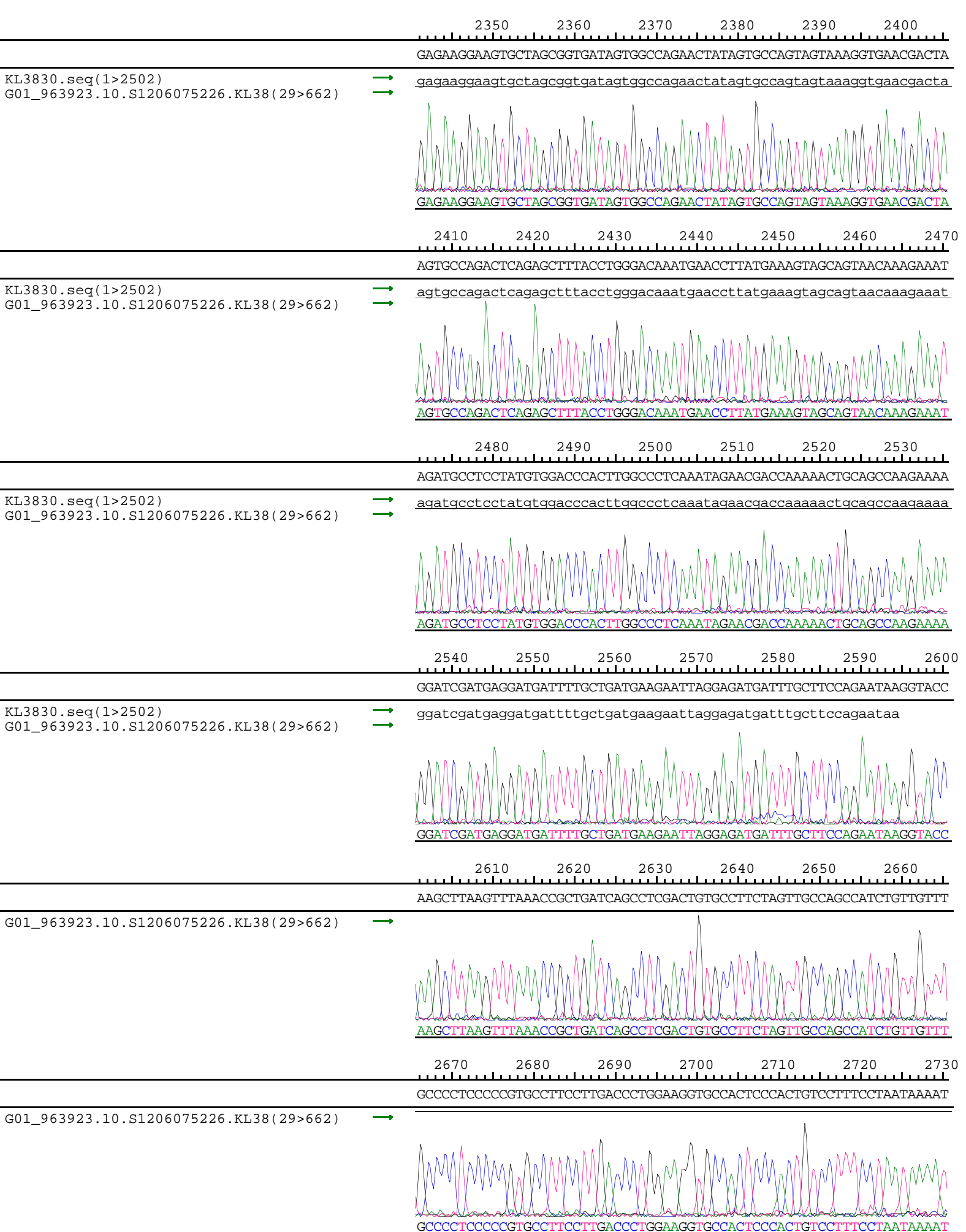

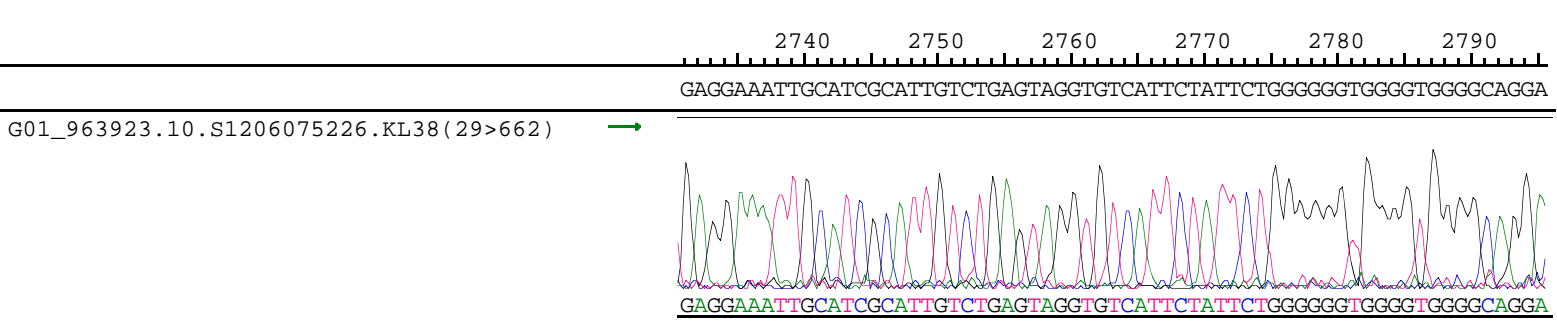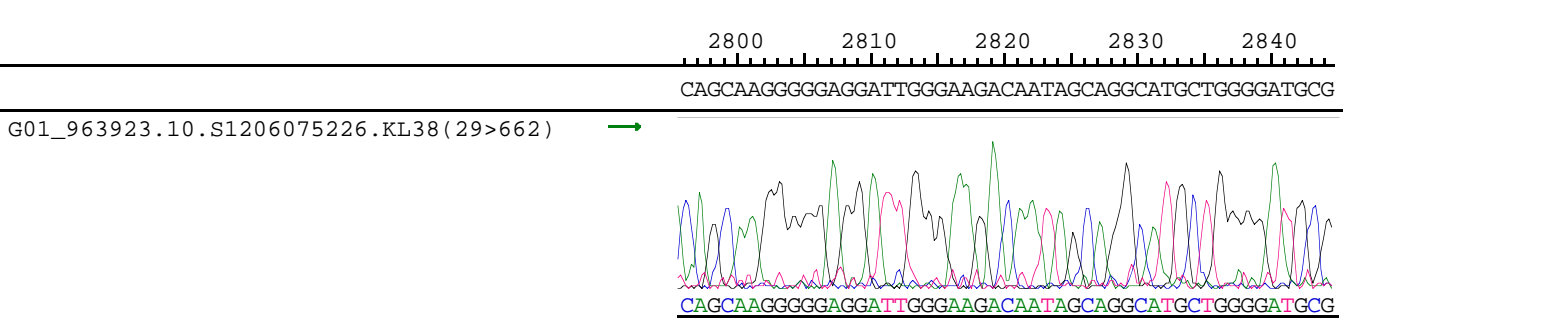

Supplement: Additional file 2 — Sequence analysis. [file 1756-9966-31-75-S2.pdf]
